# Medical Editors’ Association

**Published:** 1880-06-20

**Authors:** 


### EDITORIAL AND MISCELLANEOUS.

### *THE MEDICAL EDITORS' ASSOCIATION.*

The fifth annual session of the American Medical Editors' Association was held on Monday evening, May 31, 1880, in the parlor of the Fifth Avenue Hotel. In the absence of the president, Dr. Thos. S. Powell, of Atlanta, Georgia, editor of the Southern Medical Record, the vice-president, Dr. Frank Woodbury, of the Boston Journal, presided at the meeting. Dr. Dudley S. Reynolds, of Louisville, was appointed secretary, in the absence of Dr. Davis, of Chicago. About thirty editors were present.

Dr. Woodbury, in a short address, called the meeting to order.

The metric system was discussed informally, and a communication from Dr. Baldwin, of Columbus, opposing it, was received and read. On motion the subject was laid over until the next meeting.

Dr. A. N. Bell read a very eloquent Annual Address, sent by the President, Dr. Powell, that gentleman being unavoidably detained at home by sickness in his family.

A resolution of thanks for the address and of condolence for the illness which kept the President away, was unanimously adopted, and the resolution ordered to be transmitted to Dr. Powell.

Drs. Bell, Brodie, and Nelson were appointed Nominating Committee, who reported the following officers for 1880:

For President, Dr. George F. Schrady, of the New York Medical Record.

Vice-President, Dr. F. Nelson, of St. Louis Archives of Medicine.

Secretary, Dudley S. Reynold, of Louisville Medical Record.

Dr. Wm. Hammond, of New York, editor of Neurological Contributions, was, on motion, directed to cast the ballot of the Association, and these officers were declared unanimously elected. Place of meeting to be determined by the action of the American Medical Association, the time to be the evening before the convening of that body.—*Boston Med. and Surg. Journal.*

